# COVID-19 and Social Vulnerabilities in Virginia ZIP Code Tabulation Areas

**DOI:** 10.7759/cureus.97578

**Published:** 2025-11-23

**Authors:** Parthay H Patel, Nikhil Amin, Bhaumik Patel

**Affiliations:** 1 School of Business, Virginia Commonwealth University, Richmond, USA; 2 Department of Biology, University of Virginia, Charlottesville, USA; 3 Division of Hematology, Oncology and Palliative Care, Virginia Commonwealth University, Richmond, USA

**Keywords:** covid-19, pandemic preparedness, public health, social vulnerabilities, virginia

## Abstract

Background

We retrospectively reviewed demographic determinants (e.g., area-level ethnic and racial composition, income, and household crowding) of COVID-19 prevalence in Virginia. Data spanning May 15, 2020, to August 6, 2020, during the first COVID-19 peak in the state, were obtained from the Virginia Department of Health (VDH).

Methods

Associations between COVID-19 cases and socioeconomic variables were examined according to Virginia ZIP Code Tabulation Areas (VZCTAs) in Virginia’s health planning regions (VHPRs). Multivariate regression analysis and Pearson correlations were employed in data analyses to understand the relationship between various socioeconomic factors.

Results

The prevalence of COVID-19 was highest in the suburban Northern region and lowest in the rural-predominant southwestern region (p < 0.05). Overall, area-level ethnic and racial composition (Black/Hispanic populations), median household income, and household crowding were the most important predictors of COVID-19 prevalence in VZCTA communities in univariate and multivariate analyses, with a few important regional differences (multivariate analyses: overall R = 0.612, northern VHPR R = 0.903).

Conclusions

The study highlights area-level racial and ethnic composition, used as a proxy for structural inequalities, in the context of VHPRs as an important indicator of social vulnerability to COVID-19. The findings of this study can guide critical public health decisions, e.g., vaccine distribution or implementation of critical health policies based on social vulnerability in smaller population units.

## Introduction

COVID-19 quickly evolved into a global pandemic following its first appearance in 2019, with far-reaching impacts on the daily lives of populations. As of December 21, 2024, there were 704,753,890 cases and 7,010,681 deaths due to COVID-19 worldwide [1]. The pandemic highlighted the differential impacts on communities in the United States, particularly exposing the social vulnerabilities of certain populations [2]. Research to date has examined the contributions of demographic, socioeconomic, and host factors to COVID-19 infection in large population groups [2-4].

Certain ethnic minority groups experienced higher COVID-19 case rates, largely due to structural and social inequities. From March 1, 2020, to May 13, 2023, African Americans and Hispanic people had 1.1 times and 1.5 times higher reported COVID-19 case rates, respectively, compared with non-Hispanic White individuals [2]. Racial and ethnic disparities in COVID-19 case rates have also been reported in other groups, driven by structural factors [3]. The CDC suggests these disparities may be due to discrimination, healthcare access and utilization, occupation, economic gaps, and housing [2]. While area-level ethnic and racial composition may correlate with outcomes on a macro level, its impact on disease transmission within micropopulation units may vary.

Income and education are additional factors that may influence COVID-19 social vulnerability. Systemic inequities in educational access disproportionately affect many minority communities, contributing to differences in educational attainment and income. According to the U.S. Bureau of Labor Statistics, in 2018, workers with lower education levels were less likely to be able to work from home compared with higher-educated counterparts, limiting the ability to isolate during a pandemic [5]. Beyond education, the age composition of a micropopulation may influence population behavior. Adults aged 18-24 have been reported to show lower adherence to social distancing and mask recommendations than older adults, contributing to higher case rates [6,7]. Regarding social distancing, the CDC has also hypothesized that household crowding and poor housing conditions can increase the spread of COVID-19 [8]; however, additional studies are needed to confirm this association.

Previous studies of factors influencing COVID-19 transmission have primarily used county- or state-level data (macropopulation units). To contain a rapidly evolving pandemic in the future, a multi-pronged approach including resource management, behavioral interventions, and effective vaccine distribution is necessary. Understanding COVID-19-associated socioeconomic factors within micropopulation units, such as ZIP codes, is critical for developing effective intervention policies. ZCTA-level data groups certain ZIP codes to create standardized geographical census blocks, which facilitate statistical representation. ZCTA-level data also allow examination of local trends in disease transmission. Although ZCTAs do not cover all geographical areas, excluded areas are noted in the analysis [9].

The Virginia Department of Health (VDH) divides the state into five distinct Health Planning Regions (HPRs), each with different resource allocations. For example, over 51% of Virginia hospitals are located in the Eastern and Southwestern Regions [10]. This study examines the socioeconomic determinants of COVID-19 prevalence according to VZCTA within different HPRs to understand how demographic factors within micropopulation units influence COVID-19 transmission. The results may identify inequalities within the healthcare system and help mitigate health risks for vulnerable populations during future pandemics.

This study aims (1) to identify key socioeconomic determinants (e.g., area-level ethnic and racial composition, income, and household crowding) of COVID-19 prevalence in Virginia ZIP Code Tabulation Areas (VZCTAs) during the first COVID-19 peak (May 15 to August 6, 2020) and (2) to assess regional differences in these associations across Virginia’s five HPRs to inform public policy for future pandemics of similar scale.

This article was previously presented, in part, as a poster at the American Geophysical Union Fall 2020 meeting on COVID-19 and Earth and Space Science on December 16, 2020.

## Materials and methods

Source of data

Data were obtained from the U.S. Census Bureau (2018) American Community Survey five-year estimates and the VDH. The Census data provided demographic information for ZCTAs in Virginia, while VDH provided data on PCR testing encounters and COVID-19 cases. All data were collected from publicly accessible sources (census.gov and vdh.virginia.gov), and the corresponding raw data files are cited in the references [11,12]. COVID-19 cases were recorded over approximately three months, from May 15, 2020, to August 6, 2020.

ZIP codes in the VDH dataset were matched to their corresponding ZCTAs using a numerical crosswalk. Each ZCTA was then assigned to a county based on the county associated with its corresponding ZIP code, using zipcodestogo.com [13]. Counties were subsequently assigned to one of Virginia’s five HPRs according to official VDH designations [14]. While most ZCTAs align entirely within a single region, a smaller number may span multiple counties or cross regional boundaries. As a result, some geographic misclassifications are possible, but these are expected to be rare and unlikely to affect the regional analysis.

Variables

The study’s primary independent variables (Table 1) included median household income, median number of rooms, household size, percentage of households with more than 1.51 occupants per room, median age, percentage with a bachelor’s degree, percentage with a professional degree, percentage of Hispanic residents, percentage of African American residents, and PCR testing encounters per 100,000 people.

**Table 1:** Variable descriptions All Census variables are sourced from the U.S. Census Bureau’s 2018 American Community Survey five-year estimates. COVID-19 case and testing data were obtained from the VDH (May 15, 2020 to August 6, 2020). Variable definitions are based on the U.S. Census Bureau, and detailed explanations can be found at census.gov. VDH, Virginia Department of Health

Variable	Explanation	Source	Justification	Any alteration of data
Median household income	Median income and benefits in 2018 inflation-adjusted dollars for households in ZCTAs	Census (2018) Estimate	Study the relationship between financial factors and COVID-19 transmission.	Cases with missing values were removed. Plus signs on the data were removed.
Median room	The median number of rooms in a ZIP code community	Census (2018) Estimate	Study the relationship between crowding within the household and COVID-19 transmission	Cases with missing values were removed. Plus signs on the data were removed.
Average household size	Calculated by dividing the number of persons in households by the number of households	Census (2018) Estimate	Study the relationship between crowding within the household and COVID-19 transmission.	Cases with missing values were removed. Plus signs on the data were removed.
Percent above 1.51 occupants per room	Percent in a ZCTA, in which there are more than 1.51 occupants per room for a household, derived from the Census	Census (2018) Estimate	Study the relationship between crowding within the household and COVID-19 transmission	Cases with missing values were removed.
% Bachelor’s degree	% People who have a bachelor’s degree in ZCTAs	Census (2018) Estimate	Study the relationship between education and COVID-19 transmissions.	Cases with missing values were removed.
% Professional degree	% People who have a professional degree in ZCTAs	Census (2018) Estimate	Study the relationship between education and COVID-19 transmissions.	Cases with missing values were removed.
% Hispanic people and African Americans	% Hispanic people and % African Americans in a ZCTA	Census (2018) Estimate	Study the relationship between area-level racial and ethnic composition, specifically the proportions of African American and Hispanic residents, which were selected because prior literature consistently identifies these populations as disproportionately affected due to structural inequities and COVID-19 transmission.	Cases with missing values were removed.
PCR testing encounters	Number of PCR testing encounters per ZCTA	VDH (May 15, 2020 to August 6, 2020)	Determine PCR testing inequalities as a variable in detecting COVID-19 cases.	PCR testing encounters were converted to PCR testing per 100,000 people.
COVID-19 cases	Number of COVID-19 cases per ZCTA	VDH (May 15, 2020 to August 6, 2020)	Dependent variable	Suppressed values were estimated at 2.5 using a midpoint approximation. Zero values were removed (81 cases). Extreme outliers are removed when needed. A natural log was taken of the data. COVID-19 cases were also converted to cases per 100,000 people.

The dependent variable was COVID-19 cases per 100,000 people. This variable was adjusted for population to ensure comparability across ZCTAs of different sizes. Additionally, a natural log transformation was applied to the dependent variable. After transformation, the data were approximately normally distributed.

ZCTAs with missing values for key independent variables were excluded from the analysis using listwise deletion. No imputation techniques were applied, and exclusion of these cases did not meaningfully affect the geographic balance of the sample.

Methodology for analysis

After data collection and processing (Table 1), 759 ZCTAs were included in the analysis. Each ZCTA was assigned to an HPR, such as Northern, Northwest, Eastern, Central, or Southwest, and analyses were conducted both collectively and by region. One ZIP code (24011) was removed as an outlier for PCR testing per 100,000 individuals, as it exceeded two standard deviations above the mean. Nonsignificant variables and those exhibiting multicollinearity were excluded from the multivariate regression analysis. Additionally, the VDH suppressed case counts between 1 and 4; in such cases, a value of 2.5 was imputed to represent the average of the possible range.

Descriptive statistics, multivariate regression analysis, and Pearson correlations were employed for data analysis. The natural log of COVID-19 cases per 100,000 individuals was used as the dependent variable to linearize relationships. Significant differences in descriptive statistics were assessed using the Kruskal-Wallis test with Bonferroni correction against the Northern HPR, as a nonparametric approach was necessary due to non-normality. Pearson correlation tests were conducted to determine the significance of correlations between demographic factors and the natural log of COVID-19 cases per 100,000 individuals within each region. All bivariate tests were two-tailed.

A multivariate regression analysis was then performed to identify the most significant combination of effects and create a comprehensive model. Assumptions of multicollinearity, independence of errors, heteroscedasticity, and normality of residuals were evaluated. Variance inflation factor values were below 3, indicating no multicollinearity issues. Using the Durbin-Watson statistic, independence of errors was confirmed for all regions except the Eastern Region. Heteroscedasticity was assessed via plots of standardized residuals against predicted values, showing no significant patterns. Normality of residuals was evaluated using the Shapiro-Wilk test, which indicated non-normality; however, due to the large sample size, analysis proceeded. These minor assumption violations may limit the precision of model estimates and should be interpreted with caution.

All analyses were conducted on all Virginia ZCTAs and individually by HPR. Data analysis was performed using IBM SPSS Statistics for Windows, Version 29.0.2.0 (Released 2024; IBM Corp., Armonk, NY, USA).

## Results

Descriptive statistics 

Table 2 presents the descriptive statistics of variables for each of Virginia’s HPRs (VHPRs). First, the median values of each COVID-19 predictor were analyzed across the 759 ZCTAs in Virginia. The data were then analyzed according to their respective HPR: Northern Region (91 ZCTAs), Northwest Region (172 ZCTAs), Eastern Region (157 ZCTAs), Central Region (139 ZCTAs), and Southwest Region (200 ZCTAs).

**Table 2:** VHPRs descriptive statistics ^*^ indicates P < 0.05 using the Kruskal-Wallis test, as determined by the Kruskal-Wallis test with Bonferroni correction, comparing all groups to the Northern Region. Median values are displayed. HPR, Health Planning Region; VHPR, Virginia’s Health Planning Region; ZCTAs, ZIP Code Tabulation Areas

Variable	Total (Virginia)	Northern Region	Southwest Region	Central Region	Eastern Region	Northwest Region
Number of ZCTAs in HPRs	759	91	200	139	157	172
COVID-19 cases of 100,000 in ZCTA	669	1017	420^*^	694^*^	923	679^*^
PCR testing/100,000 individuals in ZCTA	11,341	12,295	7833^*^	13,816	14,034	10,466^*^
Median household income in ZCTA	56,972	123,791	43,910^*^	54,476^*^	56,250^*^	65,733^*^
Median rooms per household in ZCTA	5.9	6.6	5.5^*^	5.9^*^	5.9^*^	6.2
Percent of individuals with a bachelor’s degree in ZCTA	14.40%	31.90%	10.4%^*^	13.3%^*^	14.6%^*^	15.1%^*^
Self-identified % Hispanic and African American in ZCTA	16.20%	21.20%	4.45%^*^	31.90%	30.70%	9.7%^*^
Median age in ZCTA	43.3	37.2	45.75^*^	43.4^*^	43^*^	43.7^*^

The Northern Region had substantially higher median income, median number of rooms, and percentage of residents with a bachelor’s degree or higher, suggesting a higher socioeconomic status compared with other HPRs. The Northern Region population was also relatively younger and experienced the highest number of COVID-19 cases per capita. Rates of COVID-19 PCR testing in the Northern Region were generally similar to other regions, except for the Southwest and Northwest regions.

The Southwest Region differed notably from other regions, with the lowest COVID-19 case counts and PCR testing rates. Additionally, the Southwest Region had the oldest population, the lowest educational attainment, and the lowest percentages of African American and Hispanic residents.

Pearson correlation matrix

Table 3 presents unadjusted Pearson correlations between various demographic factors and the natural log of COVID-19 cases per 100,000 individuals. The correlations indicate a weak positive relationship between income, percentage of households with more than 1.51 occupants per room, and educational attainment with COVID-19 case rates. Median age showed a weak negative correlation with COVID-19 cases. The percentages of Hispanic and African American residents exhibited a moderate positive correlation with COVID-19 case rates.

**Table 3:** Strength of the relationship between demographic factors and COVID-19 cases ^*^ indicates P < 0.05 using the Pearson correlation coefficient (weak: 0.25<|r|<0.5, moderate: 0.5<|r|<0.75, and strong: 0.75<|r|<1).

Variable	Total (Virginia)	Northern Region	Southwest Region	Central Region	Eastern Region	Northwest Region
Hispanic and African American Population (%)	0.492^*^	0.768^*^	0.265^*^	0.395^*^	0.463^*^	0.154^*^
PCR testing/100,000	0.473^*^	0.773^*^	0.265^*^	0.38^*^	0.585^*^	0.145
Median household income	0.109^*^	-0.455^*^	-0.094	-0.19^*^	-0.331^*^	0.091
Median room	0.005	-0.434^*^	-0.093	-0.151	-0.115	0.106
Household size	0.052	0.049	-0.208^*^	-0.221^*^	0.008	0.077
Percent above 1.51 occupants per room	0.137^*^	0.56^*^	0.114	0.04	-0.129	0.101
Median age	-0.194^*^	-0.275^*^	0.101	-0.122	-0.178^*^	-0.192^*^
% Bachelor’s degrees	0.114^*^	-0.536^*^	-0.007	-0.03	-0.33^*^	0.086
% Professional degrees	0.126^*^	-0.48^*^	-0.038	0.034	-0.099	-0.057

Subdivisions based on HPRs showed varying results and significant influences on COVID-19 transmission. For example, the Northern Region exhibited more significant influences than the Northwest Region. This suggests that demographic factors in some regions may have a greater impact on COVID-19 transmission, potentially due to interactions between these factors within each unique environment.

Multiple regression analysis

For Virginia overall, the significant predictors were median income and area-level ethnic and racial composition (% Hispanic and % African American residents). Accounting for mediating or moderating variables, the results suggest that areas with higher proportions of Hispanic and African American residents, groups historically disproportionately affected by structural inequities, experienced higher COVID-19 case rates.

Median income appeared to mediate the effect of educational attainment, meaning that the percentage of residents with a bachelor’s degree was not a significant predictor in the analysis. Similarly, median income may have mediated the effects of household crowding (percent above 1.51 occupants per room) and median age, as partial correlations for these variables approached zero. Although these variables were not statistically significant, they may still be relevant for future studies due to factors such as small effect size or high variance.

The results varied substantially across HPRs. The Northern Region had multiple significant predictors, whereas the Northwest Region had none. The model R-value for the Northern Region was 0.903 (strong correlation), while other regions had much lower R-values, such as the Southwest Region (R = 0.376) and Northwest Region (R = 0.278). These findings indicate that demographic factors in the Northern Region, which has higher income, higher educational attainment, and a younger population, may have exerted a stronger influence on COVID-19 transmission compared with other regions.

Table 4 presents these findings.

**Table 4:** Multivariate analysis of major COVID-19 predictors (partial correlations) ^*^ indicates P < 0.05 using multiple regression. Pearson correlation coefficient: weak: 0.25<|r|<0.5, moderate: 0.5<|r|<0.75, and strong: 0.75<|r|<1. The dependent variable (COVID-19 cases per 100,000) was log-transformed, and the values represent part correlations, which represent the unique contribution of each variable to COVID-19 incidence after controlling for the effects of other variables in the model.

Variable	Total (Virginia)	Northern Region	Southwest Region	Northwest Region	Eastern Region	Central Region
Hispanic and African American Population (%)	0.314^*^	0.263^*^	0.205^*^	0.028	0.283^*^	0.293^*^
PCR testing/100,000	0.321^*^	0.380^*^	0.178^*^	0.123	0.5^*^	0.297^*^
Median household income	0.079^*^	0.113^*^	-0.07	0.029	-0.021	-0.076
Percent above 1.51 occupants per room	0.047	0.125^*^	0.027	0.092	-0.118^*^	-0.027
Median age	-0.027	-0.032	0.164	-0.151^*^	-0.059	-0.009
% Bachelor’s degrees	0.021	-0.008	0.041	0.07	-0.077	0.149^*^
Model R value	0.612	0.903	0.376	0.278	0.733	0.527
Adjusted R²	0.369	0.802	0.114	0.044	0.519	0.245

## Discussion

Understanding COVID-19-associated socioeconomic factors within micropopulation units, such as ZCTAs, is critical for developing effective intervention policies in future pandemics. The results demonstrate how certain demographic characteristics in defined micropopulation units are more likely to be affected during public health crises. COVID-19 data can be extrapolated to inform other pandemic response measures. These findings can guide critical public health decisions, such as vaccine distribution or implementation of essential health policies based on social vulnerability within smaller population units, ZCTAs. It should be noted that the results of this study are most applicable to the early phases of the COVID-19 pandemic in Virginia.

Descriptive statistics, multivariate regression analysis, and Pearson correlations effectively analyzed data for Virginia ZCTAs and each HPR. The analyses were conducted using IBM SPSS Statistics for Windows, Version 29.0.2.0. This approach is well-suited for the study. Other researchers have employed odds ratios [15] and structural equation modeling (SEM) analysis [16], although SEM is conceptually similar to multiple regression. The methodology used in this study for analyzing demographic factors and COVID-19 is consistent with previous research [12,13,16]. However, this study uses ZCTAs as the unit of analysis, whereas most other studies rely on county- or state-level data. ZCTAs, as smaller geographic units, allow for a more precise understanding of transmission within immediate communities, avoiding issues of homogeneity within counties and enabling detection of disparities at a finer scale. Statistical significance (two-tailed p < 0.05) served as the primary criterion for analysis validity.

The study highlights that area-level racial and ethnic composition functions as a proxy for systemic inequities contributing to social vulnerability for COVID-19, modified by other factors regionally. Housing characteristics and income were significant contributors to COVID-19 cases in the urban Northern HPR but not in the rural Southwestern HPR. Previous research supports the notion that areas with higher proportions of Hispanic and Black individuals experienced higher reported COVID-19 cases early in the pandemic, reflecting structural inequities and differential access to resources [4]. Limited access to disease prevention information and healthcare disparities may contribute to these trends. For example, previous literature reports that African Americans are more likely to experience delays in care due to waiting times and transportation barriers compared with other ethnic groups [14]. Addressing healthcare inequities is essential to preventing disease spread at larger scales during pandemics.

Moreover, studies have shown that the COVID-19 pandemic revealed larger disparities in mortality rates among ethnic groups, such as Hispanic and African American populations, compared with the 1918 influenza pandemic [15]. These findings underscore the importance of monitoring ethnicity-related structural inequities as indicators of social vulnerability. Disease prevention strategies can be optimized for communities disproportionately affected by such inequities, ensuring resources reach areas of greatest need.

This study has several methodological limitations. First, socioeconomic data were drawn from 2018 Census estimates, which carry inherent margins of error and may not fully reflect conditions during the 2020 pandemic due to temporal mismatch. Second, the Census Bureau’s suppression of outliers led to the exclusion of some data points. Third, the VDH did not assign a significant number of COVID-19 cases and PCR testing encounters to corresponding VZCTAs, potentially affecting results. Additionally, VDH suppressed counts between 1 and 4, requiring the imputation of 2.5 to represent the average of the possible range. The exclusion of zero values and suppressed cases may introduce bias, particularly in disproportionately rural or low-prevalence areas.

Testing availability may also differ by income, with higher-income individuals more likely to be tested, while lower-income communities may experience underreporting due to limited access [16]. Lastly, the study period (May 15, 2020, to August 6, 2020) captures only the first pandemic peak, limiting generalizability to later phases. Future studies with longer observation periods are warranted.

Despite these limitations, the study can inform Virginia’s initial governmental responses to future pandemics by identifying region-specific socioeconomic and demographic differences to guide targeted resource allocation. While prior studies have explored similar topics, preemptive research can enhance disease prevention strategies by accounting for demographic factors that increase risk due to structural inequities [17,18]. Although this study highlights important associations for pandemic transmission, these findings should not be interpreted as evidence of direct causality. Future research can validate and expand upon these results.

Importantly, while racial and ethnic composition was a significant predictor in the model, this variable reflects long-standing structural inequities rather than biological differences; race should not be pathologized. Race is a social and political construct, and observed disparities primarily reflect structural inequities.

Examining differences among locations, such as HPRs in this study, can help governments identify trends and patterns in vulnerable and nonvulnerable populations. This information enables the prioritization of preventative healthcare for marginalized groups. Health authorities should emphasize education and vaccination for populations most affected by future pandemics. Overall, the findings support a proactive, methodical approach to managing future pandemics in Virginia by preventing health issues in populations most vulnerable to disease transmission.

## Conclusions

This study highlights the importance of socioeconomic factors in influencing COVID-19 transmission across VHPRs. Our analysis identified median income, area-level racial and ethnic composition, and household crowding as key predictors of COVID-19 transmission. Among these, area-level racial and ethnic composition, the proportion of Hispanic and African American populations within a ZCTA, emerged as the most significant variable. The study also suggests that household crowding and income disparities may increase the risk of virus transmission; however, these findings require further investigation, as results varied across regions. Overall, the findings can be considered hypothesis-generating and provide a foundation for future research.

Significant regional variations were observed across VHPRs. Notably, the Northern VHPR, which is comparatively more affluent, exhibited a stronger correlation between demographic factors and COVID-19 prevalence. These regional differences underscore the need for tailored public health interventions that account for the unique characteristics of each region. Ultimately, this study offers insights that can help public health authorities develop more targeted and equitable interventions to protect populations disproportionately affected by social vulnerabilities during the early stages of a pandemic in Virginia.
